# Alleviative Effect of Iodine Pretreatment on the Stress of *Saccharina japonica* (Phaeophyceae, Laminariales) Caused by Cadmium and Its Molecular Basis Revealed by Comparative Transcriptomic Analysis

**DOI:** 10.3390/ijms241914825

**Published:** 2023-10-02

**Authors:** Xuemei Wang, Tifeng Shan, Shaojun Pang

**Affiliations:** 1 Chinese Academy of Sciences and Shandong Province Key Laboratory of Experimental Marine Biology, Center for Ocean Mega-Science, Institute of Oceanology, Chinese Academy of Sciences, Qingdao 266071, China; wangxuemei@qdio.ac.cn; 2 Laboratory for Marine Biology and Biotechnology, Qingdao National Laboratory for Marine Science and Technology, Qingdao 266071, China

**Keywords:** *Saccharina japonica*, iodine, transcriptome, cadmium stress, vHPOs

## Abstract

Iodide is accumulated by the brown alga *Saccharina japonica* at a high concentration and has been proven to be an inorganic antioxidant that plays an important role in oxidative metabolism. Vanadium-dependent bromoperoxidases (vBPOs) and iodoperoxidases (vIPOs), which catalyze the oxidation of iodide, are essential for iodine accumulation and metabolism. Heavy metal pollutant cadmium (Cd) from anthropogenic activities can cause damage to algae mainly by producing oxidative stress. Here, the effects of iodine pretreatment on the stress of *S. japonica* caused by cadmium were analyzed. The growth experiment showed that iodine pretreatment could reduce the damage of low concentration cadmium on *S. japonica* young thalli. At the transcriptomic level, gene ontology (GO) enrichment analysis confirmed that cadmium stress could cause a peroxidation reaction in *S. japonica*. However, the most significant GO term was “photosystem I” in the series with iodine pretreatment. Weighted gene co-expression network analysis (WGCNA) indicated that iodine pretreatment alleviated cadmium stress responses of *S. japonica* by affecting the photosynthesis process. Analysis of the differentially expressed genes (DEGs) showed that five enzymes from the vBPO family and 13 enzymes from the vIPO family might play crucial roles in this process.

## 1. Introduction

*Saccharina japonica* is one of the strongest accumulators of iodine among marine macroalgae, with almost 5‰ iodine in its thallus, in an organic or inorganic state. Compared with red algae and green algae, brown algae such as Laminariales (kelps) are more likely to enrich iodine. In general, the iodine contents are 10–100 μmol (g dw)^−1^ for Laminariales, 1–10 μmol (g dw)^−1^ for Fucales, 0.1–1 μmol (g dw)^−1^ for Rhodophyta, and 0.1 μmol (g dw)^−1^ for Chlorophyta [1]. Iodine metabolism in kelp is an important part of the biogeochemical iodine cycle and has important ecological significance [2]. The accumulated iodide in kelp can scavenge a variety of reactive oxygen species (ROS) and is regarded as an inorganic antioxidant. Upon oxidative stress, iodide in kelp reacts with hydrogen peroxide (H_2_O_2_) to produce hypoiodous acid (HIO), catalyzed by vanadium-dependent haloperoxidases (vHPOs). Hypoiodous acid can further react with iodide to produce volatile iodine molecules which can be released to the surface of algae, thus reducing the content of ROS in cells [3].

The brown alga *Saccharina japonica*, generally inhabiting cold water regions, is the most consumed commercial seaweed in China. Its annual production was 1742, 378 t in 2021, with Fujian being the major farming province [4]. *S. japonica* can be used as a food and also as a raw material for the extraction of bioactive chemicals such as iodine, alginate, and fucoidan [5,6]. The content of iodine in seawater can affect the growth and development of kelp. For example, Yan et al. [7] found that high concentrations of iodide (100 ppm) could affect the development of female gametophytes in *S. japonica*, inhibit sporophyte formation, and promote the formation of asexual germ lines. However, it is still unknown whether seawater iodine content has effects on the antioxidant capacity of kelp.

In the aquatic ecosystem, cadmium (Cd) is one of the most common heavy metal pollutants, which mainly results from anthropogenic activities. Heavy metal pollution can cause serious ecological risks to algae populations, fishery populations, their habitats, and even the whole marine ecosystem [8]. In *Sargassum fusiforme*, cadmium stress negatively affects metabolic activity by regulating the enzymes involved in carbohydrate and energy metabolism [9]. In *S. japonica*, cadmium was found to inhibit some protein synthesis and lead to enzyme deficiency [10]. Research has shown that cadmium stress results in lipid peroxidation and increased H_2_O_2_ content in the seaweed *Nannochloropsis oculata* [11]. Plants usually employ some processes such as uptake, translocation, sequestration, and detoxification to dispose of heavy metal stress [12]. Heavy metals may generate free radicals and ROS, and thus produce oxidative stress in living organisms.

Vanadium-dependent haloperoxidases (vHPOs), which catalyze the oxidation of halide ions, are essential for halogen metabolism in algae [13]. Activities of vHPOs in brown algae, especially kelp, may be related to their extraordinary iodine accumulation ability. The vHPO family consists of chloroperoxidases (vCPOs), bromoperoxidases (vBPOs), and iodoperoxidases (vIPOs), according to the catalyzed halides [14]. Among them, vBPOs and vIPOs can catalyze the reaction between iodide and ROS [15], probably playing a role in the detoxification of some forms of damage related to peroxidation. vHPOs are key enzymes involved in the iodine metabolism of kelp.

It is known that excess cadmium may generate free radicals and ROS and produce oxidative stress in *S. japonica*, and that iodide can protect kelp from peroxidation-induced damage. However, whether a higher iodine content can relieve the damage caused by Cd stress remains unknown. The objectives of the present study were to (i) analyze the effects of iodine pretreatment on *S. japonica* stress caused by cadmium through comparing growth and photosynthetic parameters and (ii) to explore the mechanisms of iodine pretreatment affecting the cadmium resistance of kelp from a transcriptomic perspective.

## 2. Results

### 2.1. Effects of Cadmium and Iodine on Growth of S. japonica Sporophytes

**Figure 1:**
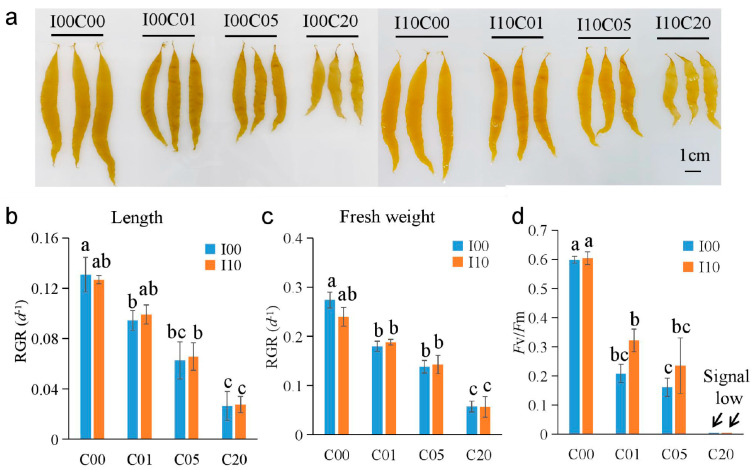
Comparison of morphological and physiological properties of *Saccharina japonica* sporophytes exposed to different concentrations of cadmium (C00, C01, C05, C20 for 0, 1, 5, 20 mg L^−1^ groups) for five days after 10 mg L^−1^ iodine pretreatment for 48 h (I10) or no pretreatment (I00). (**a**) Morphological characteristics. (**b**) Relative growth rate (RGR) for length. (**c**) RGR for fresh weight. (**d**) Maximum quantum yield of photosystem II (*F*_v_/*F*_m_). The data are the mean of triplicates. Vertical bars indicate standard deviations. Different letters indicate statistically significant differences (*p* < 0.05).

On the fifth day of cadmium stress, *S. japonica* sporophytes of all exposure groups were smaller than those of the control group of the corresponding series (Figure 1a). The blades from two high concentration groups (I00C20 and I10C20) were obviously corrugated, and the meristems (the basal section) were much narrower than those from the control groups. Regarding the blade lengths from the I00 series, the RGRs from C01, C05, and C20 were significantly lower than those from the control group (Figure 1b). Regarding the I10 series, the RGR from C20 was significantly lower than that from the control group and the RGRs from groups C01 and C05 were a little lower than those from the control group, but the differences were not significant. Regarding fresh weight, the results of the RGRs exhibited the same trend as those of the lengths (Figure 1c). The differences in the RGRs (for length and fresh weight) among I10C01, I10C05, and I10C00 were less than those among I00C01, I00C05, and I00C00. This showed that iodine pretreatment reduced the damage from low concentration cadmium on *S. japonica* young thalli. On the fifth day of cadmium treatment, the *F*_v_/*F*_m_ of C01 and C05 were significantly lower than those of group C00 in both the I00 and I10 series (Figure 1d). The *F*_v_/*F*_m_ values of two high concentration groups (I00C20 and I10C20) were too low to be detected by PAM.

### 2.2. Quantification of Iodine and Cadmium in Saccharina japonica Thalli

On the fifth day of cadmium stress, the iodine contents of *S. japonica* thalli in the I10 series were much higher than those in the I00 series (Table 1). For the I00 series, there was no significant difference in iodine content among different groups. The iodine contents of C05 and C20 were significantly higher than those of C00 and C01 in the I10 series. The cadmium contents of *S. japonica* thalli in different groups significantly increased with an increase of cadmium concentration in the culture media. However, there was no significant difference in cadmium content between the I00 and I10 series at the same experimental concentration of cadmium.

### 2.3. Trend Analysis

In the I00 series (the order was I00C00, I00C01, I00C05, I00C20), the number of trend genes was 4258, and they were clustered to 20 profiles, with eight of them being significant profiles (*p* ≤ 0.05, Figure S1a). The gene numbers of profile 0 (continuous decrease) and profile 19 (continuous increase) were 850 and 520, respectively. In the I10 series (the order was I10C00, I10C01, I10C05, I10C20), the number of trend genes was 4123, and they were clustered to 20 profiles, with eight of them being significant profiles (Figure S1b). The gene numbers of profile 0 (continuous decrease) and profile 19 (continuous increase) were 273 and 366, respectively.

**Figure 2:**
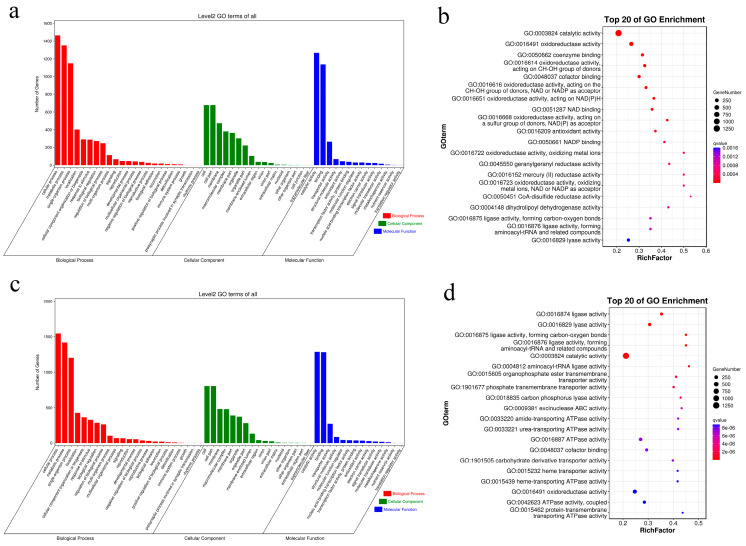
Gene ontology (GO) analysis of trending genes under increased concentrations of cadmium stress after iodine pretreatment (I10) or no pretreatment (I00). (**a**) GO analysis of 4258 trending genes in the I00 series. (**b**) GO analysis of the trending genes in the I00 series according to *p*-values. (**c**) GO analysis of 4123 trending genes in the I10 series. (**d**) GO analysis of the trending genes in the I10 series according to *p*-values.

**Figure 3:**
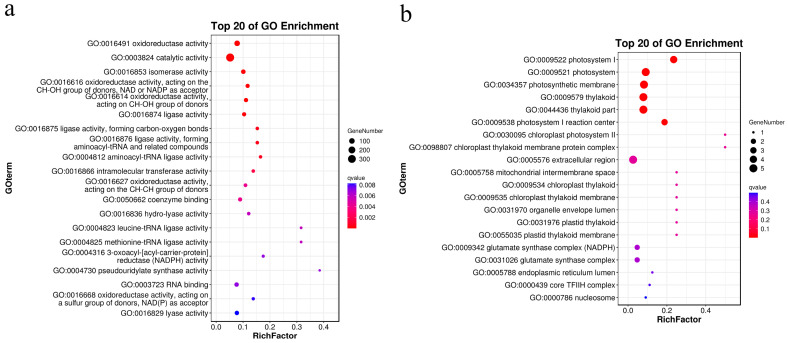
Gene ontology (GO) analysis of down-regulated genes (profile 0) under increased concentrations of cadmium stress after iodine pretreatment (I10) or no pretreatment (I00). (**a**) GO analysis of 850 down-regulated genes (profile 0) in the I00 series. (**b**) GO analysis of 273 down-regulated genes (profile 0) in the I10 series.

In the I00 and I10 series, gene ontology (GO) enrichment analysis showed that “cellular process”, “metabolic process”, “single-organism process”, “localization”, and “cellular component organization or biogenesis” were the most enriched biological process GO terms, while “cell”, “cell part”, “membrane”, “macromolecular complex”, and “membrane part” were the most enriched cellular component terms. In the I00 series, “catalytic activity”, “binding”, “transporter activity”, “structural molecule activity”, and “antioxidant activity” were the most enriched molecular function terms (Figure 2a). In the I10 series, “catalytic activity”, “binding”, “transporter activity”, “structural molecule activity”, and “molecular function regulator” were the most enriched molecular function terms (Figure 2c). In the GO enrichment bubble chart, we found that three terms were related to “oxidoreductase activity” among the six most significant terms in the I00 series (Figure 2b), and three terms were related to “ligase activity” among the five most significant terms in the I10 series (Figure 2d). In addition, we found that three terms were related to “oxidoreductase activity” among the five most significant terms in profile 0 of the I00 series (Figure 3a), and three terms were related to “photosystem” among the five most significant terms in profile 0 of the I10 series (Figure 3b).

### 2.4. Analysis of the Differentially Expressed Genes (DEGs)

**Figure 4:**
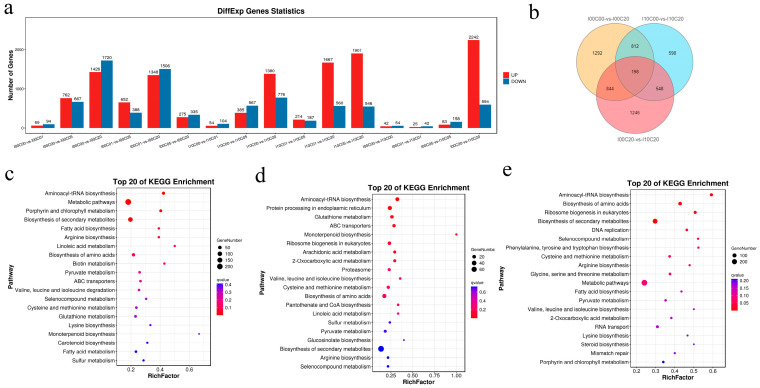
Differentially expressed genes (DEGs) among different treatment groups. (**a**) Number of DEGs identified in pairwise comparisons. (**b**) Venn diagram showing the common and specific DEGs in the three representative couples. (**c**) Kyoto encyclopedia of genes and genomes (KEGG) analysis of DEGs between I00C00 and I00C20. (**d**) KEGG analysis of DEGs between I10C00 and I10C20. (**e**) KEGG analysis of DEGs between I00C20 and I10C20.

Intra-series and inter-series pairwise comparisons were conducted to identify DEGs among samples. The number of DEGs was highest in I00C20-vs-I00C00 (HP1, 3146: 1426 up- and 1720 down-regulated), which was higher than that in I10C20-vc-I10C00 (HP2, 2156: 1380 up- and 776 down-regulated) (Figure 4a). In the inter-series comparison, the number of DEGs in I10C20-vs-I00C20 (HP3, 2836: 2242 up- and 594 down-regulated) was higher than in other pairs. The numbers of DEGs shared between the pairwise comparisons were 812 (HP1 and HP2), 844 (HP1 and HP3), and 548 (HP1 and HP3) (Figure 4b). The number of DEGs shared by these three comparisons was 198. The numbers of DEGs specific to HP1, HP2, and HP3 were 1292, 598, and 1246, respectively.

Kyoto encyclopedia of genes and genomes (KEGG) pathway enrichment analysis of DEGs in HP1, HP2, and HP3 was performed (Figure 4c–e). The response pathways shared between HP1 and HP2 were “linoleic acid metabolism”, “ABC transporters”, “glutathione metabolism”, and “monoterpenoid biosynthesis”; those shared between HP1 and HP3 were “metabolic pathways”, “porphyrin and chlorophyll metabolism”, “fatty acid biosynthesis”, and “lysine biosynthesis”; and those shared between HP2 and HP3 were “ribosome biogenesis in eukaryotes”, “2-oxocarboxylic acid metabolism”, and “valine, leucine and isoleucine biosynthesis”. The response pathways shared by these three comparisons were “aminoacyl-tRNA biosynthesis”, “biosynthesis of secondary metabolites”, “arginine biosynthesis”, “biosynthesis of amino acids”, “pyruvate metabolism”, “selenocompound metabolism”, and “cysteine and methionine metabolism”.

### 2.5. DEGs Related to Iodine Metabolism

The transcription of several vHPO genes was shown to be regulated. Five vBPOs, eight vIPOs, and five putative vIPOs were found in the DEGs of HP1, HP2, and HP3 (Table 2). Among them, a new cadmium-inducible vBPO (MSTRG.19135) was characterized. Both vBPOs and vIPOs are related to iodine metabolism, catalyzing the reaction between iodide and reactive oxygen species (ROS). The values of log_2_(fc) ranged from *−*5.2 to 13.2 for five vBPOs and seven vIPOs in HP1, from *−*9.4 to 3.8 for two vBPOs and seven vIPOs in HP2, and from *−*3.7 to 2.5 for two vBPOs and six vIPOs in HP3. Two DEGs coding for vBPOs were down-regulated, and three DEGs coding for vIPOs were up-regulated in HP1 and HP2. Two DEGs coding for vIPOs were up-regulated in HP2 and HP3. One DEG coding for vBPO and two DEGs coding for vIPOs were up-regulated in HP1, but down-regulated in HP3. One DEG coding for vBPO was down-regulated in HP1, but up-regulated in HP3.

### 2.6. Weighted Gene Co-Expression Network Analysis (WGCNA)

**Figure 5:**
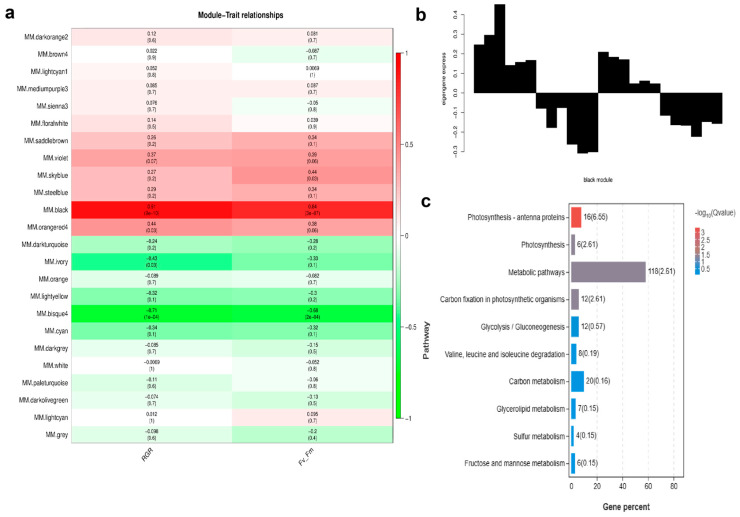
Module–trait correlations, gene expression patterns, and Kyoto encyclopedia of genes and genomes (KEGG) analysis of eigengenes in the “black” module correlated with relative growth rate (RGR) for length and maximum quantum yield of photosystem II (*F*_v_/*F*_m_). (**a**) Correlation of the modules with traits under different cadmium stresses after iodine pretreatment or no pretreatment and corresponding *p* values. Red and green colors denote positive and negative correlation, respectively. (**b**) Expression patterns of the “black” module, which highly correlated with RGR and *F*_v_/*F*_m_. (**c**) KEGG annotation of genes in the “black” module.

The WGCNA analysis resulted in 24 distinct modules. Figure S2 shows a cluster dendrogram of co-expressed genes with assigned module colors. Table S2 lists the number of genes clustered in each module. RGR and *F*_v_/*F*_m_ module–trait correlation was analyzed based on the identified modules. Figure 5a revealed that the genes in the “black” module were highly correlated with RGR (r = 0.91, *p* = 9 × 10*^−^*^10^) and *F*_v_/*F*_m_ (r = 0.84, *p* = 3 × 10*^−^*^7^). Genes in the “bisque4” module were negatively correlated with these two traits.

Figure 5b shows expression patterns of 1947 genes in the “black” module. The “black” module indicated these eigengenes were lower in the samples of higher cadmium concentration groups (I00C05, I00C20, I10C05, I10C20), but were higher in the control and low cadmium concentration groups (I00C00, I00C01, I10C00, I10C01). In the KEGG enrichment analysis of “black” module, genes were enriched in photosynthesis, carbon metabolism, and glycolysis/gluconeogenesis, etc. (Figure 5c). Genes enriched in photosynthesis–antenna proteins (16 genes) and photosynthesis (6 genes) pathways are summarized in Table S3. Their gene expression patterns for different treatments are shown in Figure S3. Of the 18 annotated genes, 15 genes belonged to the family of light harvesting complex protein (LHCP). Table S4 shows modules of DEGs involved in iodine metabolism, which revealed that two vBPOs (SJ07392, SJ10798) were clustered in the “black” module.

### 2.7. Validation of DEGs by Reverse Transcription Quantitative PCR (RT-qPCR)

**Figure 6:**
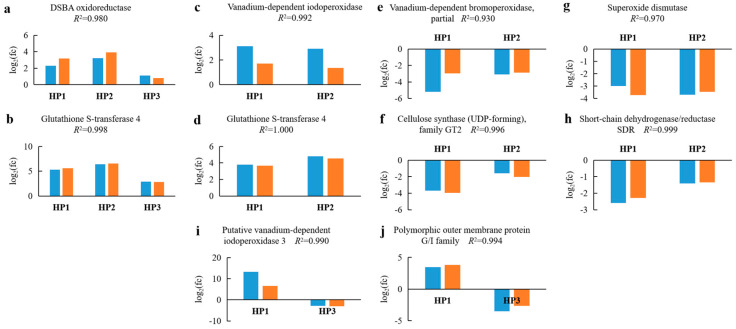
Reverse transcription quantitative PCR (RT-qPCR) validation of the expression levels of the 10 selected differentially expressed genes (DEGs) (**a**–**j**). RNA-seq and RT-qPCR data are indicated in blue and orange, respectively. HP1, HP2, and HP3 indicate I00C20-vs-I00C00, I10C20-vs-I10C00, and I10C20-vs-I00C20, respectively.

Four up-regulated unigenes coding for “DSBA oxidoreductase”, “glutathione S-transferase 4” (two transcripts), and “vIPO”; four down-regulated unigenes coding for “vBPO, partial”, “cellulose synthase (UDP-forming), family GT2”, “superoxide dismutase”, and “short-chain dehydrogenase/reductase SDR”; and two unigenes coding for “putative vIPO3” and “polymorphic Outer membrane protein G/I family” (up-regulated in HP1 and down-regulated in HP3) were randomly chosen for measuring expression levels by RT-qPCR. The fold change levels determined by RT-qPCR and RNA-seq were compared for each unigene (Figure 6). The correlation coefficients (*R*^2^) of all the selected DEGs were higher than 0.9. Therefore, the expression levels measured by RNA-seq and RT-qPCR were consistent, confirming the reliability of the RNA-seq analysis.

## 3. Discussion

Three concentration gradients (low, medium, and high concentration) of cadmium were systematically utilized to observe the influences of iodine pretreatment on *S. japonica* young thalli from the individual and transcriptome levels. The results of the RGRs and *F*_v_/*F*_m_ indicated that iodine pretreatment could relieve the harm of low concentration cadmium in *S. japonica*. In kelp, the accumulated form of iodine is iodide in an inorganic state, and iodide can scavenge different kinds of ROS, such as hydrogen peroxide and hydroxyl radicals [3]. Heavy metal stress generally can cause peroxidation reactions in live organisms. Cadmium can accumulate in *Ulva compressa* even at a low dose (1 µM), and stimulate ROS and deplete nitric oxide (NO) formation [16]. A similar reaction has also been found in the unicellular green alga *Scenedesmus quadricauda* [17]. The viability of and amount of ROS in *S. quadricauda* were negatively affected by the excessive cadmium uptake. The protective effect of iodine pretreatment on cadmium stress is probably related to the antioxidant process, but this needs further verification at the molecular level.

The results of the GO enrichment analyses of the I00 series showed that “catalytic activity” and “oxidoreductase activity” were two of the most significant terms (Figure 2b), indicating that cadmium stress can induce the stress response and establishment of the self-defense system in *S. japonica*. In the I10 series, “ligase activity” and “lyase activity” were two of the most significant terms according to the GO enrichment results (Figure 2d). There was no “oxidoreductase activity” term in the seventeen most significant terms. These results provide clues for the antioxidant activities of *S. japonica* in the iodine pretreatment groups. Besides antioxidant activities, iodide was proven to participate in osmotic regulation, supplementing that of mannitol in *Laminaria digitata* under hypersaline conditions. Moreover, iodine accumulation in *L. digitata* can positively affect photo-physiology [18]. In the present study, the three most significant terms were coincidentally related to “photosystem” in profile 0 of the iodine pretreatment groups (I10 series).

In the DEGs, a certain amount of vHPOs (mainly vBPOs and vIPOs) were annotated. These two families of enzymes can catalyze the oxidation of iodide ions with hydrogen peroxide as an oxidant. This process may play a protective role against the oxidative stress induced by excess cadmium in *S. japonica*. Among these five vBPOs and thirteen vIPOs, one vBPO (ID: MSTRG.19135, Table 1) was newly assembled, different from the others, which could be mapped to the reference genome. In the transcriptome of *S. japonica* without abiotic stress, only 5 vIPO genes were detected, and 37 vBPO genes were identified [17]. The iodine pretreatment condition likely resulted in the increase in the number of vIPOs, which were used to accumulate more iodine in the present study. vIPOs mediate iodine uptake, while vBPOs can mediate both bromide and iodine uptake. Compared with bromide, kelps prefer to accumulate iodide, which is demonstrated to be more suitable than bromide as an antioxidant against most ROS [19]. Bromide is supposed to be a supplement to the iodine antioxidant system.

Photosynthesis is the key physiological process of plant biomass formation. In the present study, the KEGG enrichment analysis of the selected module (“black”) showed that the photosynthesis process was correlated with RGR and *F*_v_/*F*_m_, the traits representing the influences of cadmium stress and iodine pretreatment on *S. japonica*. This result also gave evidence suggesting iodine pretreatment in kelp can positively affect photo-physiology [18], thus alleviating cadmium stress in *S. japonhica.* The results of trend analysis also supported this inference. Light harvesting (Table S3) and carbon fixation (Figure 5c) were the processes mainly affected in the cadmium and iodine treatment groups.

## 4. Materials and Methods

### 4.1. Algal Materials, Cultivation, and Treatment

Sporophytes of *Saccharina japonica* cultivar “E25” were generated by crossing the female gametophyte of A040 and the male gametophyte of B013 [20]. The thalli were cultured with sterilized seawater enriched with 70 mg L^−1^ NaNO_3_ and 10 mg L^−1^ NaH_2_PO_4_ at 12 °C and 50 μmol photons m^−2^ s^−1^ (fluorescent white light, 12 h light:12 h dark photoperiod) in a GXZ intelligent light incubator (Jiangnan Co. Limited, Ningbo, China). Analytically pure KI and CdCl_2_ were used for iodine and cadmium treatments, respectively. Iodine pretreatment was conducted by transferring a half quantity of the juvenile sporophytes (about 4 cm long) to culture medium plus KI with a final iodine concentration of 10 mg L^−1^ (designated as I10) for 48 h. The rest of the sporophytes with no iodine pretreatment were designated as I00. Then, the algae were cultured under concentrations of 1, 5, and 20 mg L^−1^ Cd (designated as I10C01, I10C05, I10C20 for the I10 series, and I00C01, I00C05, I00C20 for the I00 series, Table 3). The algae cultured with medium without the addition of Cd were used as controls (I00C00 and I10C00). During the experiment, the culture medium was renewed daily.

During the experiment, lengths and fresh weights of the algae were measured. The relative growth rates (RGRs) for length were calculated by the formula RGR (*d*^−1^) = (ln *L*_t_ − ln *L*_0_)/*t*, where *L*_0_ and *L*_t_ are the lengths (cm) of thalli at the initial time and after *t* days of Cd treatment. The RGRs of fresh weight were calculated by the equation RGR (*d*^−1^) = (ln*W*_t_ − ln*W*_0_)/*t*, where *W*_0_ and *W*_t_ are the fresh weights (g) of thalli at the initial time and after *t* days of Cd treatment. After 48 h of Cd treatment, three blades from each group were sampled for RNA extraction. After five days of Cd treatment, the maximum quantum yield of photosystem II (*F*_v_/*F*_m_) was measured using a pulse amplitude-modulated fluorometer (Mini PAM, Walz, Germany) after the thalli were dark-adapted for 20 min. Then, the thalli were sampled for iodine and cadmium quantitative analysis.

### 4.2. Iodine and Cadmium Quantitative Analysis

The algal samples were rinsed three times with sterilized seawater, then oven-dried at 45 °C for about 12 h until a constant weight was obtained. Then, the samples were digested according to methods described by Wang et al. [21]. Iodine and cadmium contents in *S. japonica* were detected by inductively coupled plasma mass spectrometry (ICP-MS; Agilent 7500ce, Palo Alto, CA, USA) in the presence of serial standard solutions with default parameters. All data were expressed as the mean (±SD) of triplicates. The data were analyzed by one-way analysis of variance (ANOVA) using SPSS ver. 17.0. When significant differences between means were found, a Tukey’s multiple comparison was applied.

### 4.3. RNA Preparation, cDNA Library Construction, and Sequencing

The samples were rinsed three times with sterilized seawater, then immediately frozen in liquid nitrogen and preserved at *−*80 °C for total RNA extraction. Total RNA was separately extracted from the above 24 samples with a Trizol reagent kit (Invitrogen, Carlsbad, USA) according to the manufacturer’s protocol. The concentration of RNA was checked using the NanoDrop 2000 spectrophotometer (Thermo Fisher Scientific, Waltham, MA, USA). RNA quality was determined with an Agilent 2100 Bioanalyzer (Agilent Technologies, Palo Alto, CA, USA) and RNase free agarose gel electrophoresis.

The total RNA was purified to enrich mRNA by Oligo (dT) beads. Then, mRNA was fragmented into short segments with fragmentation buffer and used for double strand cDNA (dscDNA) synthesis with random hexamer primers. The dscDNA was purified with a QiaQuick PCR extraction kit (Qiagen, Venlo, The Netherlands) and end-repaired with phosphate; poly(A) was then added and ligated to Illumina sequencing adapters. Then, the products were size-selected and PCR-amplified. Twenty-four cDNA libraries (eight treatments × three biological replicates) were established and sequenced on the Illumina HiSeq2500 by Gene denovo Biotechnology Co., Ltd. (Guangzhou, China).

### 4.4. Alignment and Annotation

The raw reads were filtered with fastp [22] by removing the reads containing adapters or more than 10% of unknown bases, and low-quality reads. Then, clean reads were mapped to the ribosome RNA (rRNA) database with Bowtie2. After removing rRNA-mapped reads, the remaining clean reads were used in the following alignment. An index of the reference genome SJ V6.2 (https://bioinformatics.psb.ugent.be/gdb/Saccharina/, accessed on 26 May 2020) was built, and paired-end clean reads were mapped to the reference genome using HISAT2.2.4 [23] with “-rna-strandness RF” and other parameters set as default. The mapped reads of each sample were assembled in a reference-based approach. For each transcription region, gene abundance was quantified by calculating the FPKM (fragment per kilobase of transcript per million mapped reads) value to eliminate the effects of gene length and amount of sequencing data.

### 4.5. Differential Expression Analysis and GO Enrichment Analysis

DESeq2 software (v1.10.1) and edgeR package (http://www.r-project.org/) were employed for pairwise differential expression analysis between different treatments. Genes with an absolute fold-change ≥ 2 and FDR (false discovery rate) < 0.05 were considered differentially expressed genes (DEGs). GO enrichment analysis of the DEGs was performed based on the hyper-geometric test [24]. GO terms with FDR < 0.05 were defined as significantly enriched in DEGs. The KOBAS software (v2.3.4) was employed to perform the KEGG pathway enrichment analysis to further understand the DEGs’ biological functions by identifying significantly enriched metabolic or signal transduction pathways. The expression patterns of DEGs were clustered using the Short Time-series Expression Miner software (STEM) [25].

### 4.6. WGCNA Analysis

Gene expression values were imported into the WGCNA (v1.47) package in R [26], using the automatic network construction function blockwise modules with default settings, except the power was set to 8. Then, module eigengenes were used for correlation analysis with RGR and *F*_v_/*F*_m_. The most relevant module corresponding to data for each phenotype was identified by calculating the Pearson correlation. For genes in the most relevant module, KEGG pathway analysis was conducted to analyze the biological functions of modules.

### 4.7. Validation of DEGs by RT-qPCR

Ten unigenes were randomly selected from the DEGs for RT-qPCR validation. Gene-specific primers were designed via Primer Premier software (v5.0) and are shown in Table S1. The PrimeScript RT Reagent Kit (with a gDNA Eraser) (Takara Bio Inc, Kusatsu, Japan) was used to generate cDNAs from the total RNAs used for transcriptome sequencing. The RT-qPCR was performed using TB GreenTM Premix Ex TaqTM II (Takara Bio Inc., Kusatsu, Japan) in a CFX96 real-time PCR system (Bio-Rad Laboratories Inc., Hercules, CA, USA) with *β*-actin as an internal control gene [27]. The PCR program was performed at 95 °C for 30 s followed by 40 cycles of 95 °C for 5 s and 60 °C for 30 s. The RT-qPCR analysis of each gene and sample was conducted in triplicate. Gene expression levels were normalized to the expression levels of *β*-actin, and relative gene expression levels were calculated according to the 2^−ΔΔCT^ method [28].

## 5. Conclusions

According to the results of the RGRs, iodine pretreatment could partly alleviate damage caused by cadmium stress in the brown algae *S. japonica*. By using the genome sequence of *S. japonica* as a reference, RNA-seq analyses were conducted to explore gene expression differences under cadmium stress with iodine pretreatment or no pretreatment in the present study. The results confirmed that cadmium stress could cause a peroxidation reaction as “oxidoreductase activity” was the most significant GO term in the I00 series at the transcriptome level. On the other hand, the most significant term in the I10 series with iodine pretreatment was “photosystem I”. WGCNA analysis indicated that iodine pretreatment alleviated the cadmium stress responses of *S. japonica* by affecting the photosynthesis process. In addition, vBPOs and vIPOs may have played crucial roles in this process.

## Figures and Tables

**Table 1:** Iodine and cadmium contents in *Saccharina japonica* on the fifth day of cadmium stress.

Group	Iodine Content (mg kg^−1^)	Cadmium Content (mg kg^−1^)
I00C00	457.73 ± 9.28 ^a^	0.33 ± 0.02 ^a^
I00C01	454.20 ± 10.14 ^a^	51.49 ± 1.48 ^c^
I00C05	466.67 ± 13.36 ^a^	148.01 ± 2.15 ^d^
I00C20	490.87 ± 6.07 ^a^	425.21 ± 4.84 ^e^
I10C00	1531.50 ± 19.99 ^b^	9.28 ± 0.32 ^b^
I10C01	1552.70 ± 37.64 ^b^	48.08 ± 2.26 ^c^
I10C05	1809.00 ± 12.13 ^c^	158.76 ± 4.64 ^d^
I10C20	2236.07 ± 24.00 ^d^	434.00 ± 4.64 ^e^

Data are expressed as mean ± SD (n = 3). Different letters in the same column indicate statistically significant differences (*p* < 0.05).

**Table 2:** Differentially expressed genes (DEGs) related to iodine metabolism.

Gene ID	Gene Description	log_2_(fc)		
		I00C20/I00C00	I10C20/I10C00	I10C20/I00C20
SJ07392	vBPO, partial	−5.2	−3.1	NS
SJ07391	vBPO, partial	−3.0	−4.9	NS
SJ10798	vBPO	−1.5	NS	1.2
SJ10789	vBPO, partial	2.5	NS	−2.0
MSTRG.19135	vBPO2	11.1	NS	NS
SJ01273	vIPO1	−1.7	NS	NS
SJ15636	vIPO	2.6	2.3	NS
SJ15637	vIPO	3.1	2.9	NS
SJ15629	vIPO	3.5	3.8	NS
SJ10612	vIPO1	8.4	NS	−3.7
SJ02183	vIPO1	NS	−9.4	NS
SJ02174	vIPO1	NS	−2.6	NS
SJ15639	vIPO	NS	NS	2.3
SJ17646	Putative vIPO3	−1.5	NS	NS
SJ12376	Putative vIPO3	13.2	NS	−2.8
SJ12374	Putative vIPO3	NS	1.3	1.6
SJ12375	Putative vIPO3	NS	2.2	2.5
SJ17646	Putative vIPO3	NS	NS	2.4

vBPO, vanadium-dependent bromoperoxidase; vIPO, vanadium-dependent iodoperoxidase; NS, no significant.

**Table 3:** Concentration settings of iodine pretreatment and cadmium stress in different groups.

Iodine Pretreatment	Concentration of Added Cadmium	Group Codes
No iodine pretreatment	0 mg L^−1^	I00C00
	1 mg L^−1^	I00C01
	5 mg L^−1^	I00C05
	20 mg L^−1^	I00C20
10 mg L^−1^ iodine pretreatment for 48 h	0 mg L^−1^	I10C00
	1 mg L^−1^	I10C01
	5 mg L^−1^	I10C05
	20 mg L^−1^	I10C20

## Data Availability

The data presented in this study are available upon request from the corresponding author.

## Supplementary Materials

The following supporting information can be downloaded at: https://www.mdpi.com/article/10.3390/ijms241914825/s1.
