# Supplementary material for: Alleviative Effect of Iodine Pretreatment on the Stress of Saccharina japonica (Phaeophyceae, Laminariales) Caused by Cadmium and Its Molecular Basis Revealed by Comparative Transcriptomic Analysis
Source: Int J Mol Sci. 2023 Oct 2;24(19):14825. doi: 10.3390/ijms241914825 (PMC10573767; doi:10.3390/ijms241914825)
Supplement: Supplementary file 1 [file ijms-24-14825-s001.zip › Figure S1.pdf]

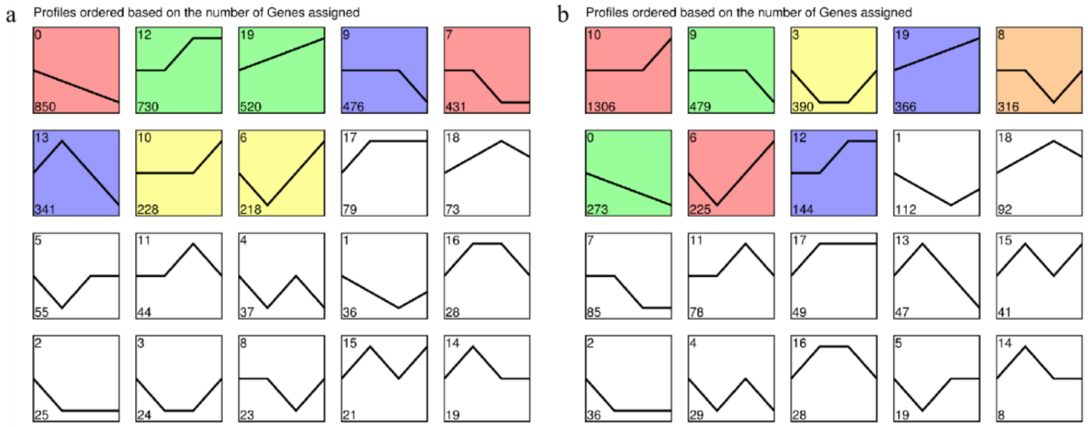


**Figure S1** Clustering results of gene expression pattern analysis in I00 series and I10 series. **a** Profiles ordered based on the number of Genes assigned in I00 series. **b** Profiles ordered based on the number of Genes assigned in I10 series. The number in the top left-hand corner of a profile box is the profile ID number. The clustered profiles with *p*-value ≤0.05 were considered as significant profiles (the colored profiles)
