# Supplementary material for: Alleviative Effect of Iodine Pretreatment on the Stress of Saccharina japonica (Phaeophyceae, Laminariales) Caused by Cadmium and Its Molecular Basis Revealed by Comparative Transcriptomic Analysis
Source: Int J Mol Sci. 2023 Oct 2;24(19):14825. doi: 10.3390/ijms241914825 (PMC10573767; doi:10.3390/ijms241914825)
Supplement: Supplementary file 1 [file ijms-24-14825-s001.zip › Table S1.pdf]

Table S1 Primers for the qRT-PCR validation

Gene ID	Forward primer	Reverse primer	Gene description
SJ07095	CAGCTGTTCCACGGGTATTT	AAGTACGGCACACCCGATAC	DSBA oxidoreductase
SJ16882	CTCCCGTGTTCCACTACCTC	GGTCGACTGGGTGAACTTGT	Glutathione S-transferase 4
SJ15637	CAGCTTCGATTTCGAGGAAC	AAAGAGGCTTGGCAAACGTA	Vanadium-dependent iodoperoxidase
SJ02439	ACCGACTACACCCAGTCCAC	CCTCGCACTTCTCCAACTTC	Glutathione S-transferase 4
SJ07392	ATGGGCAGGTGAACAAGAAC	CAGTCGTTCGGAGAGGAGAC	Vanadium-dependent bromoperoxidase, partial
SJ09104	GCCGGTTTCAAGGTGAAATA	GTAGTACAGCATGCCGCTCA	Cellulose synthase (UDP-forming), family GT2
SJ13245	GGCAGGATTCCGTACGATTA	TTCACGAAACTTTCCGTTCC	Superoxide dismutase
SJ03362	GGATAAGGGCTCGATCATCA	AAGCATAGGCTGACGGTGTT	Short-chain dehydrogenase/reductase SDR
SJ12376	ACGCTCCACAACGTCCTACT	GTCGCCTGAGACAGCATGTA	Putative vanadium-dependent iodoperoxidase 3
SJ14330	GTCATGGACTGGCGGTACTT	CGTTTCTCCGGCAAATAAAA	Polymorphic Outer membrane protein G/I family
