# Supplementary material for: Alleviative Effect of Iodine Pretreatment on the Stress of Saccharina japonica (Phaeophyceae, Laminariales) Caused by Cadmium and Its Molecular Basis Revealed by Comparative Transcriptomic Analysis
Source: Int J Mol Sci. 2023 Oct 2;24(19):14825. doi: 10.3390/ijms241914825 (PMC10573767; doi:10.3390/ijms241914825)
Supplement: Supplementary file 1 [file ijms-24-14825-s001.zip › Table S2.pdf]

Table S2 The statistics of genes in each module

Module	Gene number	percentage (%)
Ivory	11084	49.038
Bisque4	4855	21.479
Black	1947	8.614
Cyan	401	1.774
Lightcyan	356	1.575
Lightyellow	329	1.456
Darkgrey	302	1.336
Darkturquoise	302	1.336
Orange	278	1.23
White	263	1.164
Skyblue	247	1.093
Saddlebrown	232	1.026
Steelblue	224	0.991
Paleturquoise	222	0.982
Violet	206	0.911
Darkolivegreen	204	0.903
Sienna3	190	0.841
Orangered4	179	0.792
Mediumpurple3	178	0.788
Lightcyan1	159	0.703
Floralwhite	144	0.637
Darkorange2	129	0.571
Brown4	123	0.544
Grey	49	0.217
