# Supplementary material for: Alleviative Effect of Iodine Pretreatment on the Stress of Saccharina japonica (Phaeophyceae, Laminariales) Caused by Cadmium and Its Molecular Basis Revealed by Comparative Transcriptomic Analysis
Source: Int J Mol Sci. 2023 Oct 2;24(19):14825. doi: 10.3390/ijms241914825 (PMC10573767; doi:10.3390/ijms241914825)
Supplement: Supplementary file 1 [file ijms-24-14825-s001.zip › Table S3.pdf]

Table S3 Genes description in enriched pathways of photosynthesis-antenna proteins and photosynthesis from “black” module

Pathway	DEGs in pathway	Gene ID	Annotation
photosynthesis-antenna proteins	16 (6.55%)	SJ00624	LHCP
		SJ01728	LHP lhcf6
		SJ02213	LHCP
		SJ02281	LHP lhcf3, partial
		SJ02514	LHCP
		SJ02982	LHCP
		SJ03405	LHP lhcf4
		SJ04409	LHCP
		SJ05023	LHP lhcf6
		SJ06530	LHCP
		SJ07165	LHCP
		SJ09019	LHCP
		SJ09887	LHCP
		SJ12048	LHCP
		SJ13236	LHCP
		SJ17430	Fucoxanthin-chlorophyll a-c binding protein E
Photosynthesis	6 (2.61%)	SJ02433	Ferredoxin
		SJ02655	Manganese stabilising protein
		SJ06477	UN
		SJ10606	UN
		SJ15967	UN
		SJ18410	UN

LHCP, Light harvesting complex protein; LHP, Light harvesting protein; UN, Unnamed protien product
