# Supplementary material for: Alleviative Effect of Iodine Pretreatment on the Stress of Saccharina japonica (Phaeophyceae, Laminariales) Caused by Cadmium and Its Molecular Basis Revealed by Comparative Transcriptomic Analysis
Source: Int J Mol Sci. 2023 Oct 2;24(19):14825. doi: 10.3390/ijms241914825 (PMC10573767; doi:10.3390/ijms241914825)
Supplement: Supplementary file 1 [file ijms-24-14825-s001.zip › Table S4.pdf]

Table S4 Modules of differentially expressed genes (DEGs) involved in iodine metabolism

Gene ID	Gene description	Module
SJ07392	vBPO, partial	Black
SJ07391	vBPO, partial	Ivory
SJ10798	vBPO	Black
SJ10789	vBPO, partial	Ivory
MSTRG.19135	vBPO2	Bisque4
SJ01273	vIPO1	Bisque4
SJ15636	vIPO	Ivory
SJ15637	vIPO	Ivory
SJ15629	vIPO	Ivory
SJ10612	vIPO1	Bisque4
SJ02183	vIPO1	Bisque4
SJ02174	vIPO1	Cyan
SJ15639	vIPO	Ivory
SJ17646	Putative vIPO3	Ivory
SJ12376	Putative vIPO3	Ivory
SJ12374	Putative vIPO3	Ivory
SJ12375	Putative vIPO3	Ivory
SJ17646	Putative vIPO3	Ivory

vBPO, vanadium-dependent bromoperoxidase; vIPO, vanadium-dependent iodoperoxidase.
